# Clinical impact of perirenal thickness on short‐ and long‐term outcomes of gastric cancer after curative surgery

**DOI:** 10.1002/ags3.12547

**Published:** 2022-01-25

**Authors:** Kojiro Eto, Naoya Yoshida, Masaaki Iwatsuki, Shiro Iwagami, Kenichi Nakamura, Keisuke Morita, Satoshi Ikeshima, Kei Horino, Shinya Shimada, Hideo Baba

**Affiliations:** ^1^ Department of Gastroenterological Surgery Graduate School of Medical Sciences Kumamoto University Kumamoto Japan; ^2^ Department of Surgery Japan Community Health care Organization Kumamoto General Hospital Yatsushiro Japan

**Keywords:** gastric cancer, perirenal fat, predictive marker, short‐ and long‐term outcomes

## Abstract

**Background:**

A variety of factors for short‐ and long‐term outcomes have been reported after radical resection for gastric cancer (GC). Obesity and emaciation had been reported to be a cause of poor short‐ and long‐term outcomes with gastrointestinal cancer. However, the indicators are still controversial. The purpose of this study was to evaluate the relationship between perirenal thickness (PT) and short‐ and long‐term outcomes after radical surgery for GC.

**Methods:**

We analyzed the data of 364 patients with GC who underwent radical surgery. We evaluated the distance from the anterior margin of the quadratus lumborum muscle to the dorsal margin of the left renal pole using computed tomography (CT) as an indicator of PT. The association between PT and clinicopathological factors and short‐ and long‐term outcomes was evaluated.

**Results:**

The PT data were divided into low, normal, and high groups by gender using the tertile value. We found that the PT low group was 121 patients, normal group was 121 patients, and high group was 122 patients. Multivariate analyses showed that the high PT group was an independent risk factor for a short‐outcome after curative surgery in GC patients (odds ratio 2.163; 95% confidence interval [CI] 1.156–4.046; *P* = .016). And the low PT group was an independent risk factor for overall survival (hazard ratio 2.488; 95% CI 1.400–4.421; *P* = .0019) and relapse‐free survival (hazard ratio 2.342; 95% CI 1.349–4.064; *P* = .0025) after curative surgery in GC patients.

**Conclusion:**

Perirenal thickness is a simple and useful factor for predicting short‐ and long‐term outcomes after radical surgery for GC.

## Graphical abstract

This study revealed that perirenal thickness was associated with short‐ and long‐term outcomes after curative surgery in gastric cancer. Perirenal thickness is a simple and useful factor for predicting short‐ and long‐term outcomes after curative surgery for gastric cancer.

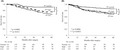

## 1 INTRODUCTION

Gastric cancer (GC) is the third‐most common cancer and the second‐most common cause of death worldwide.^1^ Radical surgery with lymphadenectomy offers a curative result for patients with resectable GC, and a variety of factors for short‐ and long‐term outcomes have been reported.

Obesity is associated with postoperative complications and worse prognosis in patients with gastrointestinal cancer, and is regarded as a major technical limiting factor for surgery because of the substantial surgical difficulties caused by abundant visceral fat (VF) and a narrow operating field. Many reports have demonstrated that obesity and abundant VF are associated with worse surgical outcomes after various surgical procedures. In these reports, obesity was associated with increased surgery time, blood loss, and postoperative complications.^2^, ^3^ Moreover, the negative impact of postoperative complications on overall survival (OS) and relapse‐free survival (RFS) after radical surgery for GC has been reported and discussed.^4^, ^5^, ^6^ Similarly, emaciation has been reported to be a cause of poor short‐ and long‐term outcomes from low nutrition and sarcopenia.^7^, ^8^, ^9^ The body mass index (BMI) is one such indicator of obesity and emaciation; however, it is controversial.^10^, ^11^, ^12^

Hiki et al previously reported that the thickness of the adipose tissue dorsal to the left kidney correlated with the amount of VF and short outcome after laparoscopic distal gastrectomy for early GC.^13^

However, there is no report on the association between long‐term outcome and perirenal thickness (PT). We hypothesized that when there is much PT, postoperative complications increase and when there is little PT, the long‐term prognosis worsens from low nutrition and sarcopenia compared to when the PT is normal. The purpose of this study was to evaluate the relationship between perirenal thickness and short‐ and long‐term outcomes after surgery for GC.

## 2 PATIENTS AND METHODS

### 2.1 Study subjects and outcomes

From January 2010 to December 2014, a total of 364 patients with GC underwent curative gastrectomy at the Department of Gastroenterological Surgery, Kumamoto University Hospital and the Department of Surgery, Kumamoto General Hospital. These patients were analyzed in this retrospective study. We excluded cases of neuroendocrine carcinoma (two cases), special types (five cases), and perioperative mortality (three cases) from this study. We retrospectively evaluated patients' perioperative characteristics, which were obtained from hospital and surgical records and included patient age, sex, BMI, comorbidities, blood test results, surgical time, blood loss, reconstruction method, and postoperative complications. Clinical staging was determined during preoperative examinations, which consisted of upper endoscopy, endoscopic ultrasonography, thoracoabdominal computed tomography (CT), barium radiography, and upper gastrointestinal endoscopic ultrasound. Pathological findings were defined accordance to the TNM classification (American Joint Committee on Cancer Staging Manual, 7th edition). The dates and causes of death were collected from follow‐up data on the basis of clinical examinations performed every 3–6 month during the 5 years after surgery. Data collection and analysis were approved by the central Scientific Review Board of both hospitals (no. 2407).

Gastrectomy with lymphadenectomy was performed according to the Japanese Gastric Cancer Treatment Guidelines for GC.^14^ In accordance with our policy, patients with clinical stage (cStage) II or III GC underwent conventional open gastrectomy with D2 lymphadenectomy, while patients with cStage I GC underwent laparoscopic gastrectomy with D1+ lymphadenectomy. Patients with pathological stage (pStage) II or III GC, excluding pT3N0 cases, received adjuvant chemotherapy.

The primary outcome was overall survival. The secondary outcomes were postoperative complications rate and RFS.

OS was defined as the interval from surgery to the date of death from any cause, or the last follow‐up in living patients. RFS was defined as the interval from surgery to either the first recurrence or death from any cause.

### 2.2 Definition of postoperative complications

The severity of postoperative complications was evaluated using the Clavien–Dindo classification system.^15^ We defined postoperative complications in this study as conditions classified as Clavien–Dindo grade II or higher, severe postoperative complications as Clavien–Dindo grade IIIa or higher, and inflammatory complications such as anastomotic leakage, pancreatic fistula, intraabdominal infection, infection at the surgical site, and pneumonia.

### 2.3 Measurement of the perirenal thickness

We evaluated the distance from the anterior margin of the quadratus lumborum muscle to the dorsal margin of the left renal pole using CT of the transverse section, 5‐mm slice, and venous phase as in our previous article.^13^ We took our measurements using the left kidney because the position of the right kidney may be influenced by the position of the liver. We decided to take our measurement at the level where the left renal vein exits the kidney, and measured the distance from the anterior margin of the quadratus lumborum muscle to the dorsal margin of the left renal pole as an indicator of PT.

To clarify the relationship between PT and short‐ and long‐term outcomes, we divided into low, normal, and high groups for PT date by using the tertile value. Since there are differences in the amount of VF between male and female, the present study was also analyzed separately for male and female.

### 2.4 Statistics

The clinicopathological characteristics and laboratory data of the two groups were compared using the chi‐squared test for categorical variables and the Mann–Whitney *U*‐test for continuous variables. *P* < .05 was considered statistically significant. In the univariate and multivariate analyses, continuous variables, including the preoperative laboratory data, surgical duration time, and amount of bleeding, were divided by the median. We used the Cox proportional hazards model to assess the effects of covariates during both the univariate and multivariate analyses; the categorical covariates are listed in Table 3. We conducted multivariate analysis using factors with *P* < .05 in the univariate analysis. All tests were analyzed using JMP software (SAS Institute, Cary, NC, USA).

## 3 RESULTS

### 3.1 Cutoff value of perirenal thickness

In males, 76 patients were in the low group (PT: 0.4–6.1), 76 patients were in the normal group (PT: 6.2–14.1), and 76 patients were in the high group (PT: 14.2–38.8). Similarly, in females, 45 patients were in the low group (PT: 0.4–3.1), 45 patients were in the normal group (PT: 3.2–5.8), and 46 patients were in the high group (PT: 5.9–26.0). In total, we found that the PT low group was 121 patients, the PT normal group was 121 patients, and the PT high group was 122 patients.

### 3.2 Patients' and baseline characteristics

In terms of the preoperative factors, there was a significantly higher number of patients who were male in the PT high group. In terms of surgical factors, there was a significantly longer surgical time, higher amount of blood loss, and higher frequency of postoperative complications (including severe and inflammatory complications) in the PT high group. However, there was no significant difference in cStage, pStage, between the groups (Table 1).

**TABLE 1:** Association between patient characteristics and perirenal thickness of the 364 gastric cancer patients

Factors	Variable	Low	Normal	High	*P*‐value
		(N = 121)	(N = 121)	(N = 122)	
Age median, years	Median (range)	68 (22–97)	64 (27–91)	70 (36–89)	.016
Body mass index median, kg/m^2^	Median (range)	21.6 (13.4–32.3)	21.4 (14.9–32.6)	23.1 (16.6–35.1)	<.001
Body mass index median, kg/m^2^	<18.5/≥18.5, <25/≥25	15/90/16	15/86/20	5/73/42	<.001
Comorbidity (%)	Present	49 (40.5)	53 (43.8)	57 (47.1)	.584
Preoperative total protein, g/dL	Median (range)	7.0 (5.4–8.3)	6.8 (5.0–8.8)	7.1 (5.1–8.0)	.850
Preoperative albumin, g/dL	Median (range)	4.1 (2.5–4.9)	4.0 (2.7–4.8)	4.2 (2.6–4.9)	.258
Clinical T, n^a^	1/2/3/4	65/15/20/20	78/10/16/16	67/16/21/17	.650
Clinical N, n (%)^a^	Present	30 (25.2)	27 (22.5)	27 (22.7)	.860
Clinical stage, n^a^	I/II/III/IV	77/24/18/1	84/21/14/1	81/25/15/0	.826
Operative approach, n	Open/Lap	72/49	52/69	72/50	.013
Operative procedure, n	DG/PG/TG/other	71/14/35/1	86/10/25/0	68/26/27/1	.030
Lymph node dissection, n	D1/D2	66/55	74/47	68/54	.540
Reconstruction	B1/RY/others	47/59/15	48/63/10	48/47/27	.071
Surgical time, min	Median (range)	273 (103–667)	295.5 (140–716)	305 (123–674)	.036
Blood loss, mL	Median (range)	149 (0–1975)	125 (0–2916)	230 (5–4510)	.0080
Postoperative complications, n (%)	Present	31 (25.6)	27 (22.3)	47 (38.5)	.014
Postoperative severe complications, n (%)	Present	15 (12.4)	14 (11.6)	36 (29.8)	<.001
Postoperative inflammatory complications, n(%)	Present	20 (16.5)	16 (13.2)	40 (32.8)	<.001
Pathological T, n^a^	1/2/3/4	59/13/24/25	69/13/19/20	63/11/31/17	.476
Pathological N, n (%)^a^	Present	48 (39.7)	40 (33.1)	42 (34.7)	.538
Pathological stage, n^a^	I/II/III	67/22/32	76/20/25	66/34/22	.121
Adjuvant chemotherapy, n (%)	Present	26 (21.5)	24 (19.8)	25 (20.7)	.951

^a^ According to the International Union Against Cancer tumor, node, metastasis classification system (7th edition).

### 3.3 Risk factors for postoperative complications

Variables that achieved a probability value of .05 in the univariate analysis were included in a subsequent multivariate analysis to identify risk factors for postoperative complications. In the multivariate analysis, high blood loss (≥155 mL) and PT high (odds ratio 2.163; 95% confidence interval [CI] 1.156–4.046; *P* = .016) were selected as independent predictive factors for postoperative complications in GC patients who underwent curative gastrectomy (Table 2). The low PT was not found to be a predictor of postoperative complications.

**TABLE 2:** Univariate and multivariate analysis of factors affecting postoperative complications in gastric cancer patients who underwent curative gastrectomy

Factors	Univariate analysis			Multivariate analysis		
	OR	95% CI	*P*‐value	OR	95% CI	*P*‐value
Age						
<75 years	1.000			1.000		
≥75 years	1.782	1.096–2.886	.020	1.568	0.924–2.663	.096
Gender						
Female	1.000			1.000		
Male	1.653	1.024–2.718	.040	1.526	0.875–2.660	.136
Body mass index						
≥18.5 kg/m^2^ <25 kg/m^2^	1.000			1.000		
<18.5 kg/m^2^	1.171	0.551–2.487	.682	1.347	0.651–2.691	.901
≥25 kg/m^2^	1.942	1.147–3.288	.014	1.072	0.565–2.034	.833
Comorbidity						
Absent	1.000					
Present	1.082	0.683–1.710	.735			
Clinical stage^a^						
I	1.000			1.000		
II/III/IV	2.059	1.279–3.331	.0030	1.524	0.812–2.858	.189
Operation time						
<295 min	1.000			1.000		
≥295 min	1.942	1.223–3.110	.0048	1.666	0.941–2.947	.080
Blood loss						
<155 mL	1.000			1.000		
≥155 mL	2.873	1.787–4.694	<.0001	2.233	1.141–4.368	.019
PT						
Normal	1.000			1.000		
Low	0.787	0.481–1.286	.338	1.198	0.634–2.264	.579
High	1.988	1.244–3.178	.0041	2.163	1.156–4.046	.016
Lymph node dissection						
D1+	1.000					
D2	1.312	0.831–2.071	.243			
Reconstruction						
Billroth I	1.000			1.000		
Others	2.465	1.502–4.147	.0003	1.860	0.961–3.601	.066
Operative approach						
Lap	1.000			1.000		
Open	1.777	1.112–2.851	.015	1.212	0.578–2.570	.604
Operative procedure						
DG	1.000					
Others	1.545	0.973–2.451	.065			

^a^ According to the International Union Against Cancer tumor, node, metastasis classification system (7th edition).

### 3.4 Survival outcomes

**FIGURE 1:**
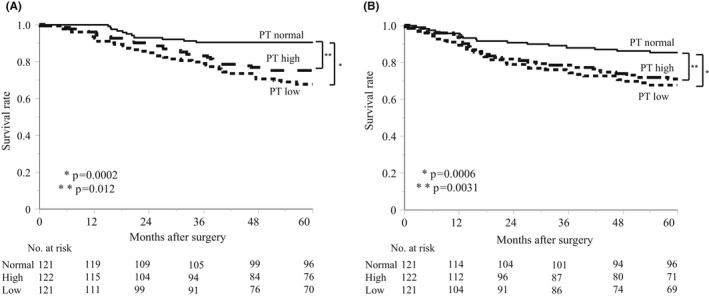
(A) Kaplan–Meier analysis of overall survival in the normal, low, and high perirenal thickness (PT) groups after resection of gastric cancer with curative intent. (B) Kaplan–Meier analysis of relapse‐free survival in the normal, low, and high PT groups after resection of gastric cancer with curative intent

The median follow‐up time was 1840 days for the entire population of 364 GC patients. The PT low and high groups had significantly poorer outcomes. The OS and RFS results in the two groups are demonstrated in Figure 1A,B, respectively. In the multivariate analysis, the PT low was selected as an independent predictive factor for OS (hazard ratio 2.488; 95% CI 1.400–4.421; *P* = .0019, Table 3) and RFS (hazard ratio 2.342; 95% CI 1.349–4.064; *P* = .0025, Table 4) in GC patients who underwent curative gastrectomy. However, PT high was not selected as an independent predictive factor for OS and RFS in this cohort.

**TABLE 3:** Univariate and multivariate analysis of factors affecting overall survival in gastric cancer patients who underwent curative gastrectomy

Factors	Univariate analysis			Multivariate analysis		
	HR	95% CI	*P*‐value	HR	95% CI	*P*‐value
Age						
<75 years	1.000			1.000		
≥75 years	3.039	1.966–4.698	<.0001	2.873	1.861–4.434	<.0001
Gender						
Female	1.000			1.000		
Male	1.867	1.163–3.109	.0090	1.531	0.947–2.475	.082
Body mass index						
≥18.5 < 25 kg/m^2^	1.000			1.000		
<18.5 kg/m^2^	1.790	0.726–4.413	.206	0.982	0.377–2.557	.970
≥25 kg/m^2^	1.948	1.258–3.014	.011	1.310	0.778–2.207	.310
Comorbidity						
Absent	1.000					
Present	1.042	0.672–1.610	.852			
Operation time						
<295 min	1.000					
≥295 min	1.032	0.661–1.605	.888			
Blood loss						
<155 mL	1.000			1.000		
≥155 mL	2.847	1.790–4.672	<.0001	1.533	0.869–2.706	.140
PT						
Normal	1.000			1.000		
Low	1.807	1.197–2.726	.0048	2.488	1.400–4.421	.0019
High	1.150	0.753–1.756	.518	1.490	0.809–2.745	.201
Lymph node dissection						
D1+	1.000			1.000		
D2	1.937	1.250–3.040	.0030	1.261	0.788–2.017	.330
Reconstruction						
Billroth I	1.000			1.000		
Others	1.879	1.173–3.114	.0081	1.103	0.577–2.110	.766
Operative procedure						
DG	1.000			1.000		
Others	1.529	1.015–2.303	.042	1.504	0.884–2.558	.133
Postoperative complications						
Absent	1.000			1.000		
Present	2.300	1.517–3.486	<.0001	1.839	1.177–2.841	.0072
Pathological stage^a^						
I	1.000			1.000		
II/III	2.603	1.671–4.127	<.0001	1.662	1.052–2.628	.030

^a^ According to the International Union Against Cancer tumor, node, metastasis classification system (7th edition).

**TABLE 4:** Univariate and multivariate analysis of factors affecting relapse‐free survival in gastric cancer patients who underwent curative gastrectomy

Factors	Univariate analysis			Multivariate analysis		
	HR	95% CI	*P*‐value	HR	95% CI	*P*‐value
Age						
<75 years	1.000			1.000		
≥75 years	2.710	1.802–4.065	<.001	2.456	1.627–3.708	<.001
Gender						
Female	1.000			1.000		
Male	1.917	1.230–3.087	.0036	1.603	1.013–2.535	.044
Body mass index						
≥18.5 < 25 kg/m^2^	1.000			1.000		
<18.5 kg/m^2^	0.481	0.196–1.183	.111	0.952	0.367–2.470	.952
≥25 kg/m^2^	2.164	1.431–3.273	.0003	1.432	0.877–2.338	.151
Comorbidity						
Absent	1.000					
Present	1.075	0.714–1.612	.728			
Operation time						
<295 min	1.000					
≥295 min	1.090	0.721–1.646	.681			
Blood loss						
<155 mL	1.000			1.000		
≥155 mL	3.034	1.962–4.826	<.001	1.445	0.841–2.483	.183
PT						
Normal	1.000			1.000		
Low	1.581	1.064–2.349	.024	2.342	1.349–4.064	.0025
High	1.307	0.877–1.947	.189	1.653	0.933–2.929	.085
Lymph node dissection						
D1+	1.000			1.000		
D2	2.264	1.500–3.463	<.001	1.444	0.923–2.260	.107
Reconstruction						
Billroth I	1.000			1.000		
Others	1.982	1.274–3.179	.0021	1.065	0.581–1.955	.838
Operative procedure						
DG	1.000			1.000		
Others	1.491	1.008–2.207	.046	1.418	0.859–2.343	.172
Postoperative complications						
Absent	1.000			1.000		
Present	2.171	1.455–3.238	.0001	1.768	1.156–2.704	.0086
Pathological stage^a^						
I	1.000			1.000		
II/III	2.643	1.749–4.054	<.001	1.677	1.088–2.585	.019

^a^ According to the International Union Against Cancer tumor, node, metastasis classification system (7th edition).

## 4 DISCUSSION

In the present study we demonstrated that PT is a simple and useful predictor for the short‐ and long‐term outcomes after curative surgery in GC patients. Our results indicated that the high PT group was associated with a higher frequency of postoperative complications, and the low PT group was associated with a potentially worsening prognosis. To the best of our knowledge, this is the first report to reveal an association between PT and short‐ and long‐term outcomes after curative surgery in GC patients.

We found that the high PT group was associated with short‐term outcomes after radical surgery for GC. BMI is widely used as an indicator of obesity in clinical settings. A higher BMI has been associated with increased complications in several studies.^3^, ^16^, ^17^, ^18^ However, another study demonstrated that there was no difference in surgical outcomes on the basis of BMI.^19^ These results show that the issue of whether BMI is correlated with short‐term outcomes remains controversial. Because BMI is calculated using body weight and height, it considers not only the VF but also the subcutaneous fat. This makes it impossible to distinguish between the two using this indicator alone. Therefore, we considered that BMI may not be suitable for measuring obesity in surgical patients and that VF volume is more indicative of obesity. However, the precise measurement of the entire VF volume requires specialized software. The method of measuring VF using CT scans and an image processing 3‐D image analysis system (eg: SYNAPSE VINCENT, DICOM images) is complex and is not suited to routine medical practice. Therefore, we focused on PT as a simple indicator of VF. This study revealed that CT assessment of PT, which was validated as a surrogate of VF, was superior to BMI for GC patients. The simplicity with which PT can be measured makes it a clinically valuable tool. Using PT, surgical teams can assess the risk of complications before surgery.

Several recent studies revealed that the occurrence of postoperative complications could have a significantly negative impact on the prognosis of patients with GC.^4^, ^5^, ^6^, ^20^ Therefore, it is important to prevent the occurrence of complications, and prediction of morbidities before surgery is considered an important task, especially in patients with malignancy. Indeed, this study revealed that postoperative complications were an independent prognostic factor after radical surgery for GC. In this study, high PT was an independent predictor of short‐term postoperative outcome, while it tended to be associated with a worse prognosis but was not an independent prognostic factor. Analysis of subgroups suggests that even high PT may not worsen the long‐term outcome without postoperative complications (Figure S1). This suggests that the high level of recruitable energy from intraabdominal fat tissue may have prevented the prognosis from worsening.^21^

We found that the low PT group was associated with long‐term outcomes after radical surgery for GC. The relationship between prognosis and the low PT content may be attributed to several factors. First, the low PT might reflect a malnutrition state. Emaciated patients may suffer from malnutrition in the early stage of postoperative body weight loss compared with normal patients. Second, VF is an energy store that correlates with physical capacity, suggesting that the low PT reserves affect cancer prognosis. Obese patients have a large energy store, which they can access in times of negative energy balance.^21^ Conversely, the basic physical capacity is much reduced in patients with low VF reserves and should present as a poor prognostic feature.^22^

Previous studies reported various systems for predicting surgical risks. The physiological and operative severity score for the enumeration of mortality and morbidity (POSSUM),^23^ modified POSSUM,^24^ and the estimation of physiological ability and surgical stress scoring system^25^ have been reported to provide reliable predictive scores for mortality and morbidity. Although these scoring systems are useful, they use both preoperative and intraoperative factors to predict postoperative morbidity and mortality. The strength of the risk prediction score proposed in the current study is that it relies solely on the preoperative risk factors. A previous study evaluated the efficacy of perioperative exercise programs in reducing postoperative complications after surgery in overweight or obese GC patients.^26^ In patients with high PT, exercise interventions, and in patients with low PT, nutritional interventions may improve not only short‐term outcomes but also long‐term outcomes after curative surgery in GC patients.

This study had several limitations. First, this study was a retrospective analysis, and thus might be subject to several biases. Second, the cutoff values used in this study may differ from those applied at other institutions; the present study analyzed continuous variables of only two institutions. Different institutions may have different cutoff values. Therefore, a prospective multi‐institutional study is desirable to validate the present findings. Interobserver reliability for these types of measurements needs to be demonstrated in further multi‐institutional studies. Third, this study only focused on the score at one point before surgery, and we also need to analyze cases with exercise and/or nutritional interventions in the future.

In conclusion, this study is the first to reveal the association between PT and short‐ and long‐term outcomes after curative surgery in GC patients. When we are able to undertake appropriate preoperative evaluations using PT, it will be possible to minimize complications to the greatest possible extent and may improve prognosis.

## DISCLOSURE

Conflict of Interest: The authors declare that they have no conflicts of interest.

Author Contributions: Study concept and design: K. Eto, N. Yoshida, and H. Baba; acquisition of data: K. Eto, N. Yoshida; analysis and interpretation of data: K. Eto, N. Yoshida; writing of the article: K. Eto, N. Yoshida, and H. Baba. All authors approved the final article.

Ethical approval:The data collection and analysis of this study were approved bythe institutional Scientific Review Board of Kumamoto University Hospital (No. 1037).

Informed consent: Informed consent was obtained from all individual participants included in the study.

## Supporting information

Figure S1
