# Supplementary material for: Clinical impact of perirenal thickness on short‐ and long‐term outcomes of gastric cancer after curative surgery
Source: Ann Gastroenterol Surg. 2022 Jan 25;6(4):496–504. doi: 10.1002/ags3.12547 (PMC9271023; doi:10.1002/ags3.12547)
Supplement: Supplementary file 1 — Figure S1 [file AGS3-6-496-s001.pptx]

## Slide 1
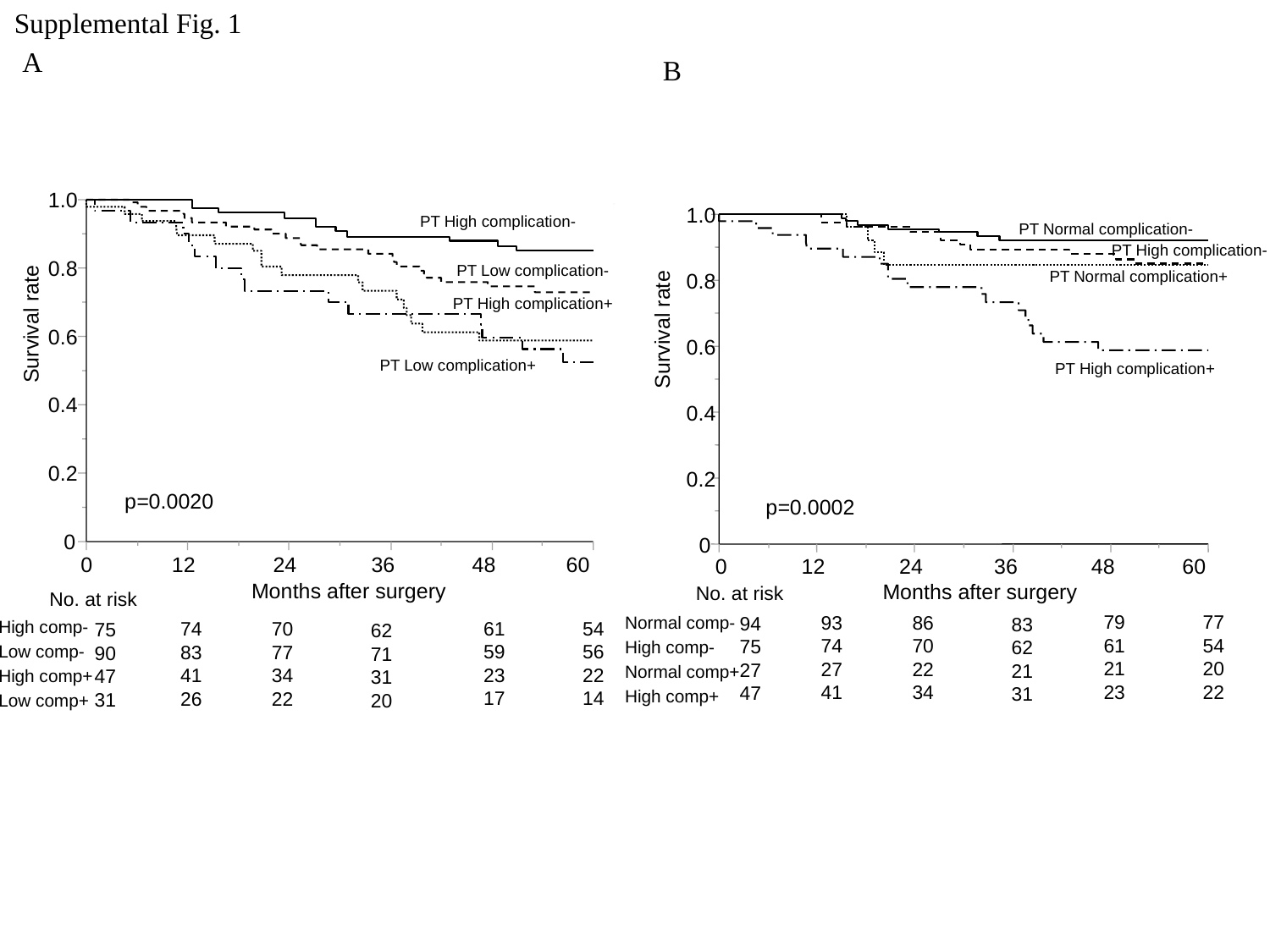

Supplemental Fig. 1
A
B
1.0
0.8
0.6
0.4
0.2
0
0
12
24
36
48
60
1.0
0.8
0.6
0.4
0.2
0
0
12
24
36
48
60
PT High complication-
PT Normal complication-
PT High complication-
PT Low complication-
PT Normal complication+
PT High complication+
Survival rate
Survival rate
PT Low complication+
PT High complication+
p=0.0020
p=0.0002
Months after surgery
Months after surgery
No. at risk
86
70
22
34
93
74
27
41
94
75
27
47
83
62
21
31
77
54
20
22
79
61
21
23
No. at risk
High comp-
Low comp-
High comp+
Low comp+
70
77
34
22
74
83
41
26
75
90
47
31
62
71
31
20
54
56
22
14
61
59
23
17
Normal comp-
High comp-
Normal comp+
High comp+
